# Novel therapeutic roles of MC‐4 in combination with everolimus against advanced renal cell carcinoma by dual targeting of Akt/pyruvate kinase muscle isozyme M2 and mechanistic target of rapamycin complex 1 pathways

**DOI:** 10.1002/cam4.1748

**Published:** 2018-08-29

**Authors:** Ji Yeon Son, Sungpil Yoon, In Hwan Tae, Yu Jin Park, Umasankar De, Yukyoung Jeon, Young Ju Park, Im Joo Rhyu, Byung Mu Lee, Kyu‐Huck Chung, Joung Eun Lim, Se Jeong Lee, Hye Won Lee, Jong Hwan Kwak, Hyung Sik Kim, Han Yong Choi

**Affiliations:** ^1^ School of Pharmacy Sungkyunkwan University Suwon Korea; ^2^ Department of Anatomy Korea University College of Medicine Seoul Korea; ^3^ Departments of Urology Samsung Medical Center Sungkyunkwan University School of Medicine Seoul Korea; ^4^ Department of Anatomy and Cell Biology Sungkyunkwan University School of Medicine Suwon Korea; ^5^ Single Cell Network Research Center Sungkyunkwan University School of Medicine Suwon Korea; ^6^ Department of Urology Kangbuk Samsung Hospital Seoul Korea

**Keywords:** *Artemisia annua* L., autophagy, everolimus, metastatic renal cell carcinoma, pyruvate kinase muscle isozyme M2

## Abstract

Current clinical trials of new anticancer therapies against metastatic renal cell carcinoma (RCC), including molecular‐targeted therapies, have not shown promise. The purpose of this study was to preclinically assess the antitumor effects of MC‐4, a partially purified material of *Artemisia annua* L., as a monotherapy or in combination with the known mechanistic target of rapamycin complex 1 (mTORC1) inhibitor, everolimus, against Caki‐1 (Von Hippel‐Lindau (VHL)+/+) and 786‐O (VHL−/−) human RCC cells. MC‐4 monotherapy significantly increased tumor growth inhibition and autophagic cell death in RCC cells *in vitro* and *in vivo*. Everolimus led to compensatory Akt activation by inhibiting only mTORC1 signaling pathway. In contrast to everolimus, MC‐4 enhanced phosphatase and tensin homolog expression and reduced its downstream effector, Akt/pyruvate kinase muscle isozyme M2 (PKM2), leading to decreased expression of glucose transporter 1, which is associated with cancer cell metabolism. The synergistic antitumor and anti‐metastatic effects induced by co‐administration of MC‐4 and everolimus involve cell growth inhibition and autophagic cell death via dual targeting of phosphatidylinositol 3‐kinase (PI3K)/Akt/PKM2 and mTORC1. These findings suggest that MC‐4 is a novel Akt/PKM2 inhibitor that can overcome the limitation of existing mTOR inhibitors and can be considered a novel strategy to treat patients with rapidly progressing advanced RCC.

## 1 INTRODUCTION

Despite the increasing number of therapeutic options,^1^ the prognosis of patients with metastatic renal cell carcinoma (RCC) at diagnosis or those with a metastatic recurrence remains dismal.^2^ Although several types of vascular endothelial growth factor‐ and mechanistic target of rapamycin (mTOR)‐targeted drugs have been approved as first‐line therapies for the treatment of metastatic RCC,^1^ more than 40% of patients do not respond to these agents.^3^ In particular, mTOR signaling pathway is a pivotal regulator of cellular growth, differentiation, survival, metabolism, and stress response.^4^, ^5^, ^6^, ^7^ mTOR complex 1 (mTORC1) phosphorylates ribosomal protein S6 kinase (S6K) and eukaryotic translation initiation factor 4E‐BP1 to modulate translation, autophagy, lipid biosynthesis, mitochondrial biogenesis, and ribosome biogenesis. mTORC2 phosphorylates serum/glucocorticoid regulated kinase 1 (SGK1), Akt, Ras‐related C3 botulinum toxin substrate 1 (Rac1), and protein kinase Cα (PKCα) to regulate cell survival, glycolytic enzymes, pentose phosphate pathway enzymes, glutaminase, and cytoskeletal organization.^4^, ^5^, ^6^, ^7^ Due to feedback between mTORC1 and mTORC2, crosstalk with other pathways leading to the compensatory activation of extracellular signal‐regulated kinase (ERK)/mitogen‐activated protein kinase pathway (MAPK),^8^, ^9^ and a higher risk of side effects, the therapeutic efficacy of FDA‐approved mTORC1 inhibitors such as everolimus is limited.^10^

Several studies have demonstrated the importance of natural products as sources of new anticancer drugs.^11^, ^12^, ^13^ For example, 47% of chemotherapeutics are of natural origin or directly derived from nature, and up to 70% are considered structurally related to natural compounds.^11^ Therefore, we focused on the discovery of novel components from natural plants, which could potentiate anticancer activities when combined with mTOR inhibitors in patients with metastatic RCC. Previously, we reported the antitumor and anti‐metastatic efficacy of artesunate, a semi‐synthetic derivative of the sesquiterpene artemisinin, against advanced RCC,^14^ consistent with other antitumor activities including anti‐angiogenesis, reversal of multidrug resistance, reactive oxygen species‐induced DNA damage, immune stimulation, and improved radiosensitivity.^15^, ^16^, ^17^, ^18^ Under the hypothesis that *Artemisia annua* L. could provide novel candidates for anticancer agents other than artemisinin,^19^ we tested the inhibitory effects of MC‐4 fraction from the aerial parts of *Artemisia annua* L. on the growth and metastasis of Caki‐1 and 786‐O human RCC cell‐lines, with the aim to identify natural materials that demonstrate effective antitumor activity against metastatic RCC, either alone or in combination with everolimus.

## 2 MATERIALS AND METHODS

### 2.1 Chemicals and reagents

Cell culture medium, fetal bovine serum (FBS), and supplements were obtained from Gibco Invitrogen Corporation (Carlsbad, CA, USA). The primary antibodies for p‐p53, p27, cyclin B1, cyclin D1, Cyclin‐dependent kinase 1 (CDK1), CDK4, B‐cell lymphoma 2 (Bcl‐2), Bcl‐2‐associated X protein (Bax2), total Poly (ADP‐ribose) polymerase (PARP), total caspase 3, p62, microtubule‐associated protein 1A/1B‐light chain 3 (LC3)‐I/II, Beclin‐1, autophagy‐related 5 (ATG5), phosphatidylinositol 3‐kinase (PI3K), phosphatase and tensin homolog (PTEN), pAkt^S473^, total Akt, pyruvate kinase muscle isozyme M2 (PKM2), p‐mTOR, total mTOR, p‐P70S6K, total P70S6K, α‐tubulin, and β‐actin were purchased from Cell Signaling Technology (Danvers, MA, USA). Anti‐Ki‐67 and anti‐Hypoxia‐inducible factor 1‐alpha (HIF‐1α) were purchased from Abcam (Cambridge, UK). Anti‐Glucose transporter 1 (GLUT1), anti‐cytochrome c, and horseradish peroxidase (HRP)‐conjugated secondary antibodies were purchased from Santa Cruz Biotechnology (Santa Cruz, CA, USA). Everolimus was purchased from Selleckchem (Houston, TX, USA). All other chemicals were purchased from Sigma‐Aldrich (St. Louis, MO, USA). Everolimus was dissolved in dimethyl sulfoxide (DMSO) and stored at −20°C until use. These agents were diluted to appropriate concentrations with culture medium containing 1% FBS. The final concentration of DMSO was less than 0.1% (v/v).

### 2.2 Extraction and fractionation of MC‐4 from *Artemisia annua* L

**Figure 1:**
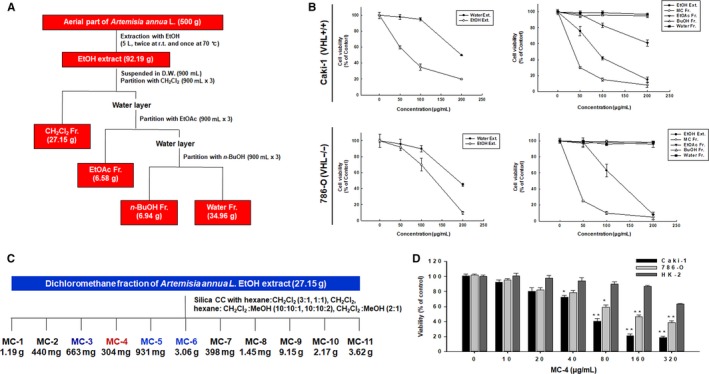
Extraction procedures of MC‐4 from *Artemisia annua* L. and its anticancer activity. A, Extraction and fractionation scheme of the aerial parts of *Artemisia annua* L. B, The cytotoxic activities of extracts and solvent fractions from *Artemisia annua* L. against two human renal carcinoma cell lines. r.t., room temperature; D.W., distilled water. C, Fractionation scheme of MC‐4 from the dichloromethane fraction of *Artemisia annua* L. ethanol (EtOH) extract. D, The anticancer activity of MC‐4 was tested in two human cancer cell lines (Caki‐1 and 786‐O) and normal human proximal tubular epithelial (HK‐2) cells. All cells were treated with MC‐4 (0‐320 μg/mL) for 24 h and cytotoxicity was measured by MTT assay (n = 3, data are expressed as mean ± SEM of triplicate experiments). CC, column chromatography. *P < 0.05, **P < 0.01

The aerial parts of *Artemisia annua* L. were collected at Yeongyang‐gun, Gyeongsangbuk‐do, Korea in July 2015. A voucher specimen (SKKU‐Ph‐15‐010) was deposited at the herbarium of the School of Pharmacy, Sungkyunkwan University. The fresh plant was dried at 25°C for 5 days (below 40% humidity). The dried aerial parts of *Artemisia annua* L. (500 g) were cut into small pieces and extracted twice with ethanol (EtOH) at room temperature (RT) for 24 hours, and once with EtOH at 70°C for 5 hours. All the extracts were combined, and the solvent was evaporated at 40°C under reduced pressure to prepare an EtOH extract (EtOH Ext., 92.19 g) (Figure 1A). The dried aerial parts of *Artemisia annua* L. (100 g) were extracted twice with distilled water at 100°C for 5 hours under reflux. The filtrate was lyophilized at −50°C for 24 hour to prepare a water extract (Water Ext., 24.99 g). The EtOH extract was suspended in distilled water (900 mL) and then sequentially fractionated with dichloromethane (CH_2_Cl_2_), ethyl acetate (EtOAc), and *n*‐butanol (*n*‐BuOH). Each of the solvent fractions was evaporated at 40°C under reduced pressure to prepare dichloromethane (CH_2_Cl_2_ Fr., 27.15 g), ethyl acetate (EtOAc Fr., 6.58 g), *n*‐butanol (*n*‐BuOH Fr., 6.94 g), and water (Water Fr., 34.96 g) fractions. Of these fractions, the dichloromethane fraction was subjected to column chromatography on a silica gel column (230‐400 mesh, Merck, Darmstadt, Germany) by stepwise elution with hexane‐CH_2_Cl_2_ (3:1 and 1:1), CH_2_Cl_2_, hexane‐CH_2_Cl_2_‐MeOH (10:10:1 and 10:10:2), and CH_2_Cl_2_‐MeOH (2:1) to produce ten subfractions (MC‐1 to MC‐10). MC‐4 fraction was obtained from the eluted portion with hexane‐CH_2_Cl_2_ (1:1). The subfraction MC‐4, which showed the highest cytotoxicity against Caki‐1 and 786‐O RCC cell lines, was characterized by gas chromatography–mass spectrometry (GC/MS) analysis (Hewlett Packard HP 6890 series GC system with a Hewlett Packard 5973 mass selective detector) (Figure 6A,B). The components corresponding to five major peaks in GC chromatogram for MC‐4 were isolated and identified from spectroscopic data. MC‐4 fraction was subjected to silica gel column chromatography. To yield five compounds, the selected fractions were rechromatographed on silica gel, RP‐C_18_, Sephadex LH‐20, and preparative thin‐layer chromatography using different solvent combinations.

### 2.3 Cell lines and culture conditions

Human RCC (Caki‐1 and 786‐O) cell lines and HK‐2 cells, an immortalized proximal tubule epithelial cell line from normal adult human kidney, were purchased from the American Type Culture Collection. Authentication of these cell lines was conducted by short tandem repeat profiling to exclude cross‐contamination between the cell lines. The RCC cells were maintained in Roswell Park Memorial Institute (RPMI)‐1640 medium supplemented with 10% FBS, 100 U/mL penicillin, and 100 μg/mL streptomycin at 37°C in a humidified atmosphere of 5% CO_2_ and 95% air. HK‐2 cells were cultured in 75‐cm^2^ plastic flasks with complete culture medium [a 1:1 mixture of Dulbecco's modified Eagle's medium (DMEM)/F12 media containing 10 ng/mL epidermal growth factor, 1% penicillin/streptomycin, 1% l‐glutamine, 15 mmol/L hydroxyethyl piperazineethanesulfonic acid (HEPES), 50 mmol/L hydrocortisone, 5 μg/mL insulin, 5 μg/mL transferrin, and 50 nmol/L sodium selenite] at 37°C with 5% CO_2_ in a humidified atmosphere.

### 2.4 Cytotoxicity assays

Cell viability was determined using 3‐(4,5‐dimethylthiazol‐2‐yl)‐2,5‐diphenyltetrazolium bromide (MTT, 5 mg/mL, Sigma‐Aldrich) assay. The cultures were seeded in 96‐well plates at a density of 2 × 10³ cells per well. After incubation for 24 hours, the cells were treated with varying concentrations of samples, and cultured for 24, 48, or 72 hours. Then, 15 μL of MTT reagent was added to each well, and incubated for 3 hour at 37°C in the dark. The supernatant was aspirated, and the formazan crystals were dissolved in 150 μL of DMSO at 37°C for 15 minutes with gentle agitation. The absorbance per well was measured at 540 nm using the Versa‐Max Microplate Reader (Molecular Devices Corp., Sunnyvale, CA, USA). Data were analyzed from three independent experiments and then normalized to the absorbance of the wells containing medium only (0%) and untreated cells (100%). The half maximal inhibitory concentration (IC_50_) values were calculated from sigmoidal dose–response curves with *SigmaPlot* 10.0 software.

### 2.5 Evaluation of apoptosis/necrosis by flow cytometry

Caki‐1 and 786‐O cells were treated with MC‐4 or everolimus at a concentration above the IC_50_ value for the RCC cell lines (100 g/mL) or with medium alone (control), and incubated for 24 hours at 37°C. After treatment, apoptosis was detected using an Annexin V‐FITC Kit (Beckman Coulter, Indianapolis, IN, USA). Both attached and detached cells were harvested, washed with phosphate‐buffered saline (PBS), and resuspended in 500 μL of ice‐cold binding buffer. The cell suspension (5 × 10^5^ cells in 100 μL) was transferred to flow cytometry tubes, and 10 μL of Annexin V‐FITC and 20 μL of propidium iodide (PI) were added. After 15 minutes of incubation in the dark, 400 μL of binding buffer was added to each tube, and the samples were analyzed using a flow cytometer (Guava^®^ EasyCyte flow cytometer, EMD Millipore, Billerica, MA, USA). The percentage of early apoptotic, late apoptotic, and necrotic cells was determined using Flowing Software (http://www.flowingsoftware.com/), and the results are shown as dot plots.

### 2.6 Monodansylcadaverine (MDC) incorporation assay

Exponentially growing cells were cultured on coverslip attached to the dish for 48 hours and then incubated with the indicated drug treatment in DMEM containing 1% FBS for 48 hours. After washing the cells with cold PBS, they were fixed with 3.75% paraformaldehyde in PBS. Then, the autophagic vacuoles were labeled with MDC, an autofluorescent base capable of accumulating in autophagic vacuoles, by incubating the cells with 0.05 mmol/L MDC at RT for 30 minutes. After incubation, the cells were visualized under a fluorescence microscope at 600× magnification (Olympus FV10i, Tokyo, Japan).

### 2.7 Transmission electron microscopy (TEM)

For sample preparation, the cells and tumor tissues were fixed in 2.5% glutaraldehyde in PBS (pH 7.4). The samples were further fixed with 1% osmium tetroxide for 1 hour, gradually dehydrated with EtOH gradients, and embedded in epoxy resin. For TEM analysis, sections (70 nm) were cut using a Leica Ultra‐CUT ultramicrotome (Leica Microsystems GmbH, Wetzlar, Germany) and contrasted with 0.1% lead citrate and 8% uranyl acetate in 50% EtOH. Ultrathin sections were examined using a transmission electron microscope (Tecnai™ G^2^ Spirit, FEI Company, Hillsboro, OR, USA) that operated at 120 kV. Images were captured using a Megaview III CCD camera (Soft Imaging System, Lakewood, CO, USA).

### 2.8 Cell cycle analysis

Caki‐1 and 786‐O cells were cultured with everolimus, MC‐4, or medium alone (control). After 24 hours, both attached and detached cells were harvested, washed in PBS, and fixed in 70% ice‐cold EtOH overnight at 4°C. The fixed cells were pelleted, resuspended in 1 mL PBS with RNAse A (500 μg/mL), and incubated for 30 minutes at 37°C. After addition of 5 μL of staining solution (10 mg of PI in 1 mL of PBS), the cells were incubated for 15 minutes in the dark. The cells were analyzed using a flow cytometer (Guava^®^ EasyCyte flow cytometer). DNA content was determined using Flowing Software, and data on cell cycle distribution were represented as histograms.

### 2.9 Western blot analysis

Caki‐1 and 786‐O cells were cultured with 100 μg/mL MC‐4, 1 μmol/L everolimus, or 0.1% DMSO in medium (vehicle control). The cells were harvested by trypsinization and washed twice with cold PBS. For isolation of total proteins, the cells were suspended in PRO‐PREP protein extraction solution (iNtRON, Seongnam, Korea). Protein concentrations were measured using a protein assay kit (Bio‐Rad, Hercules, CA, USA) according to the manufacturer's instructions. The cell extract containing 20 μg of protein was loaded onto 6%‐15% gradient polyacrylamide gels. After sodium dodecyl sulfate polyacrylamide gel electrophoresis (SDS‐PAGE), the gels were transferred onto a polyvinylidene difluoride (PVDF) membrane (Millipore, Billerica, MA, USA). The membrane was blocked with 5% skim milk, and subsequently incubated with various primary antibodies at 4°C overnight. After washing the membrane for 1 hour with TNA buffer, the membrane was incubated with horseradish peroxidase (HRP)‐conjugated anti‐mouse or anti‐rabbit antibodies (1:10 000) for 1 hour at RT. The blots were developed using an enhanced chemiluminescence (ECL) Plus kit (Amersham Biosciences, Buckinghamshire, UK).

### 2.10 *In vivo* antitumor activity in subcutaneous xenografts

Four‐week‐old female BALB/c nude mice weighing approximately 20 g (Japan SLC Inc., Hamamatsu, Shizuoka, Japan) were housed under controlled temperature (22 ± 2°C) and a 12‐hour light/dark cycle in laminar flow cabinets with filtered air. The mice were handled using aseptic procedures. The experimental procedure was approved by the Institutional Animal Care Committee of Sungkyunkwan University (SKKU2016120046). Caki‐1 and 786‐O cells (5 × 10^5^ cells/0.1 mL) in serum‐free medium containing 50% Matrigel™ (BD Biosciences, Franklin Lakes, NJ, USA) were injected subcutaneously. The mice were randomized to four groups (n = 5 per group): (a) everolimus treatment (oral gavage, 10 mg/kg); (b) MC‐4 treatment (oral gavage, 200 mg/kg); (c) treatment with both everolimus (oral gavage, 10 mg/kg) and MC‐4 (oral gavage, 200 mg/kg); and (d) treatment with 0.2 mL PBS vehicle. All the treatments were performed 5 days a week for 30 days. Tumor volume (*V*) was calculated using the standard formula, *V* (mm^3^) = 0.52 (*ab* ^2^), where *a* is the length and *b* is the width of the tumor. Body weight was recorded before drug administration and at the time of experiment termination. On the 30th day, the mice were euthanized by carbon dioxide asphyxiation.

### 2.11 Evaluation of anti‐metastatic activity using experimental lung metastasis assays *in vivo*

For the establishment of *in vivo* RCC experimental lung metastatic tumor models, Caki‐1 or 786‐O cells [2 × 10^6^ cells/0.1 mL Hank’s Balanced Salt Solution (Gibco Invitrogen Corporation, Carlsbad, CA, USA)] were injected intravenously to five‐week‐old female BALB/c nude mice (Japan SLC Inc.) through the tail vein. The mice were randomized to four groups (n = 7 per group): (a) control (oral gavage, 0.2 mL PBS vehicle); (b) everolimus treatment (oral gavage, 10 mg/kg); (c) MC‐4 (oral gavage, 200 mg/kg); and (d) combination treatment with everolimus (oral gavage, 10 mg/kg) and MC‐4 (oral gavage, 200 mg/kg). All the treatments were performed 5 days a week for 33 days from the day following the injection day. Body weight was recorded before tail vein injection until termination of the experiment. On the 34th day, all mice were euthanized by carbon dioxide asphyxiation. Their lungs were resected and fixed in 10% neutral buffered formalin for hematoxylin and eosin staining. Pulmonary nodules were counted microscopically.

### 2.12 Immunohistochemistry (IHC)

After the tumor sections were incubated with 3% hydrogen peroxide (H_2_O_2_) for 10 minutes, they were blocked with 3% goat serum containing 0.3% Triton™ X‐100 and incubated overnight with anti‐Ki‐67 (1:250) and PKM2 (1:250) monoclonal antibodies. After reaction with a biotinylated goat anti‐mouse secondary antibody, the sections were treated with HRP‐streptavidin (Santa Cruz Biotechnology, Dallas, TX, USA). The sections were then analyzed using an UltraVision™ detection system with 3,3′ Diaminobenzidine (DAB) Plus substrate (Thermo Fisher Scientific Inc., Waltham, MA, USA). Photomicrographs of the tumor sections were captured at 200× magnification using a Zeiss Axiophot light microscope equipped with a PAX‐it camera (PAXcam, Villa Park, IL, USA).

### 2.13 Quantification of glutamine and lactate levels

Caki‐1 and 786‐O cells were seeded at a density of 1 × 10^6^ cells per 25‐cm^2^ culture flask (Corning) and allowed to grow overnight. The culture media were then replaced with fresh complete RPMI media plus 6 mmol/L glucose, and the cells were treated with drugs for 24 hours. To measure the lactate and glutamine concentrations in the conditioned media, the media were centrifuged and the supernatants (50 μL) were subjected to high‐performance liquid chromatography (HPLC) analyses. Briefly, a Sunchrom C18 column (250 × 4.6 mm, 5 μm particle size, 300 Å; The Great Eur‐Asia Sci‐Tech Development Co., Ltd, Beijing, China) was attached to a HEWLETT PACKARD series 1100 HPLC system that was pre‐equilibrated in 20 mmol/L triethylamine [pH 6.5 (adjusted with phosphoric acid)] and 0.1% acetonitrile. After loading, the column was washed with starting solvent for 5 minutes and resolved with an acetonitrile gradient at a flow rate of 0.5 mL/min. The peak ultraviolet traces (260 nm) were quantified by plotting against standard curves.

### 2.14 Statistical analyses

Data are expressed as the means + standard error of mean (SEM) of at least three independent experiments. Depending on the data distribution, statistical evaluation was performed using Student's *t* tests for paired observations or using one‐way analysis of variance. The data distributions were evaluated for normality using the Shapiro‐Wilk test. Tumor volumes were analyzed by Kruskal–Wallis test, followed by Dunn's test. *P *<* *0.05 was considered statistically significant.

## 3 RESULTS

### 3.1 MC‐4 exhibits cytotoxicity against human RCC cells by inducing cell cycle arrest, apoptosis, and autophagic cell death

To identify novel anticancer compounds against RCC, we isolated fractions from the extracts of *Artemisia annua* L. (Figure 1A) that exhibited potent cytotoxic activity in human RCC lines [Caki‐1 (wild type von Hippel‐Lindau) (VHL) and 786‐O (VHL null with constitutive HIF activity)] (Figure 1B). The cytotoxicity of MC‐4 was higher than that of MC‐3, MC‐5, and MC‐6 (Figure 1C). MC‐4 significantly reduced the viability of RCC cells in a concentration‐dependent manner (Figure 1D, Caki‐1: IC_50_ = 95 μg/mL, 786‐O IC_50_ = 124 μg/mL). Normal human renal proximal tubular epithelial (HK‐2) cells were more resistant to the cytotoxic effects of MC‐4.

**Figure 2:**
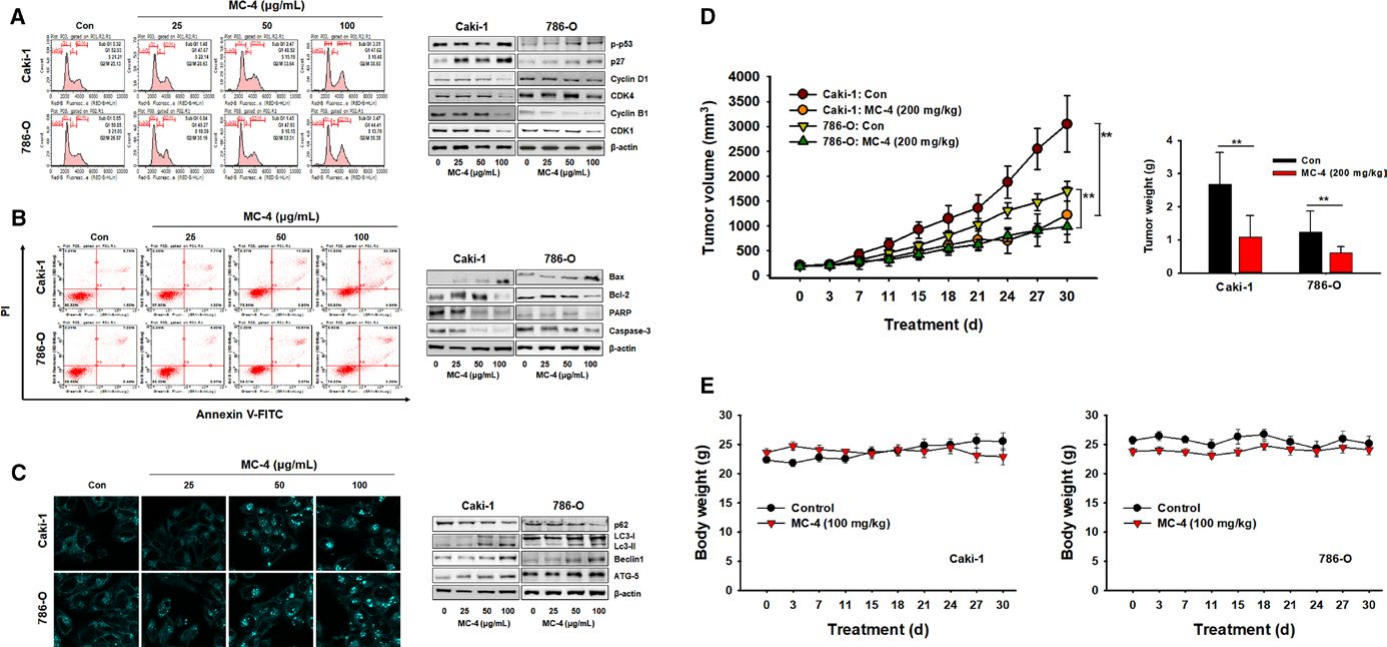
Anticancer effects of MC‐4 in human RCC cells. A, Effects of MC‐4 on the cell cycle. Caki‐1 and 786‐O cells were treated with MC‐4 (25, 50, or 100 μg/mL) for 24 h. After incubation, the cells were stained with propidium iodide (PI) and then analyzed using a flow cytometer (left panel). The expression levels of cell cycle‐regulated proteins were measured by Western blot analysis (right panel). B, Effects of MC‐4 on apoptosis. Scatter plots present the percentage of viable, early apoptotic, late apoptotic, and necrotic cells in untreated (control) and treated cells (left panel). The cells were treated with MC‐4 (25, 50, or 100 μg/mL) for 24 h. The expression levels of apoptosis‐related proteins were measured by Western blotting (right panel). C, Effects of MC‐4 on autophagy. Monodansylcadaverine (MDC) staining shows that autophagy was activated in Caki‐1 and 786‐O cells after treatment with MC‐4. Magnification ×400 (left panels). The expression levels of autophagy‐related proteins in RCC cells were measured after MC‐4 treatment. Western blot analysis was performed using p62, beclin‐1, LC3‐I/II, and ATG5 antibodies. Equal loading and transfer were verified by reprobing the membranes with β‐actin antibody (right panels). D, Effects of MC‐4 on tumor growth in nude mice inoculated with Caki‐1 or 786‐O cells. During the 30‐d treatment, tumor volumes were estimated using measurements taken with calipers (mm^3^). The histogram data shown are average tumor volumes and tumor weights (mean ± SEM, n = 5). **P < 0.01 vs vehicle control. E, Effects of MC‐4 on body weight changes in nude mice inoculated with Caki‐1 (left) and 786‐O (right) cells

**Figure 3:**
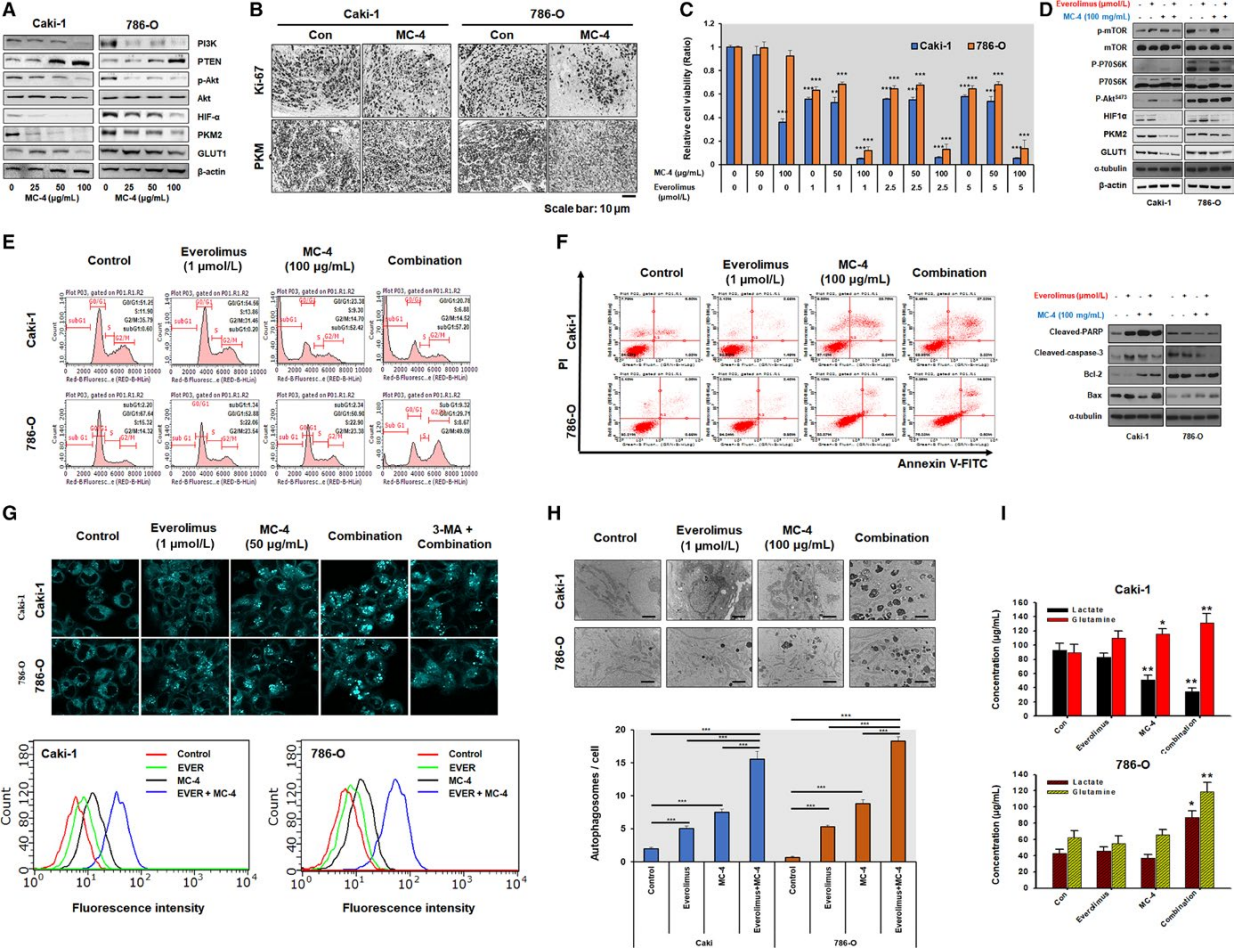
Synergistic mechanisms of anticancer activities induced by MC‐4 and everolimus alone and in combination. A, Effect of MC‐4 on the anticancer signaling pathways in RCC cells. Caki‐1 and 786‐O cells were treated with MC‐4 for 24 h, and then Western blotting was performed. Representative blots are shown. B, Representative photomicrographs of immunohistochemical (IHC) analysis of PKM2 (bottom) in the indicated tumors from mice treated with MC‐4 or vehicle (lower panel). C, Combination effects of MC‐4 and everolimus on cell viability. RCC cell lines (Caki‐1 and 786‐O) were treated for 72 h with MC‐4 in combination with everolimus. Cell viability was determined by MTT assay and is expressed relative to the value without treatment. The data are means ± SEM from three independent experiments. **P < 0.01, ***P <* *0.001. D, Signaling pathways and proteins affecting cell metabolism after treatment with MC‐4 and everolimus alone or in combination in RCC cells. Caki‐1 and 786‐O cells were treated with the indicated concentrations of MC‐4 and everolimus for 24 h, and then Western blot analyses were performed. Representative blots are shown. E, Effects of MC‐4, everolimus, and their combination on cell cycle progression. Caki‐1 and 786‐O cells were treated with the indicated concentrations of everolimus, MC‐4, or their combination for 24 h. The cells were then stained with propidium iodide (PI) and analyzed using a flow cytometer. F, Effects of everolimus, MC‐4, or their combination on apoptotic cell death. Caki‐1 and 786‐O cells were treated as indicated for 24 h, and the cells were stained with annexin V/PI and analyzed by flow cytometry. Scatter plots (left) present the percentage of viable, early apoptotic, late apoptotic, and necrotic cells in untreated (Con) and treated cells. Differential expression of apoptosis‐related proteins was measured by Western blotting (right). Representative images are shown. G, Effects of everolimus, MC‐4, or their combination on autophagy. Monodansylcadaverine (MDC) staining in Caki‐1 and 786‐O cells after treatment with the indicated compounds. The cells were examined by a fluorescence microscope. Magnification ×400 (top panels). Autophagy was analyzed by flow cytometry (bottom panels). H, Transmission electron microscopy (TEM) of RCC cells after treatment with the indicated compounds. Significant increase in number of autophagosomes was observed with combination treatment (top images). Histograms show the means ± SEM of the number of autophagosomes per cell in Caki‐1 and 786‐O cells from two experiments. **P <* *0.01, ***P <* *0.001. I, Effect of everolimus, MC‐4, or their combination on cellular metabolite levels in RCC cells. Histograms show the means ± SEM from two experiments. *P <* *0.05, **P <* *0.01

For elucidating the mechanisms underlying the MC‐4‐dependent cytotoxic effects in Caki‐1 and 786‐O cells, we first tested cell cycle distribution using flow cytometry because it is known that mTOR regulates cell cycle progression.^20^ MC‐4 induced potent G2/M cell cycle arrest of Caki‐1 and 786‐O cells (Figure 2A, left panel) by upregulating p27^Kip1^ and phospho‐p53 and downregulating cyclin B1 and CDK1/4 (Figure 2A, right panel). MC‐4 treatment increased late apoptosis in both cell types in a concentration‐dependent manner. The effect was greater in Caki‐1 cells (Figure 2B, left panel), consistent with increased Bax2 and decreased Bcl‐2, total PARP, and caspase 3 in both cells (Figure 2B, right panel). Furthermore, as shown in Figure 2C, there was significant autophagic vesicle formation in both cells exposed to MC‐4, with marked conversion of LC3‐I to LC3‐II and increase in ATG5 and beclin‐1 levels, suggesting that MC‐4 induced autophagic cell death associated with apoptosis in human RCC cells regardless of *VHL* gene mutation status. Consistent with the *in vitro* antitumor effects of MC‐4 on human RCC cells, MC‐4 (200 mg/kg, 5 days per week for 30 days) significantly inhibited tumor growth in both Caki‐1 and 786‐O subcutaneous xenografts compared to the control group (Figure 2D). No signs of toxic side effects were observed during treatment as assessed by monitoring of body weights (Figure 2E). Highly upregulated PTEN and reduced phosphorylation of Akt, PKM2, and GLUT1 expression were observed in both cell lines (Figure 3A). Consistent with the inhibitory action of MC‐4 on PKM2 expression *in vitro*, significant reduction in PKM2‐positive cells was observed *in vivo* by MC‐4 treatment (Figure 3B), indicating that MC‐4 induces autophagy against RCC possibly through the inhibition of cancer cell glucose metabolism modulated by Akt/PKM2.

### 3.2 MC‐4 combined with everolimus displays synergistic anticancer activities in targeting human RCC cells via induction of cell cycle arrest and activation of autophagic cell death

The lack of complete response to everolimus in patients with relapsed or relapsed/refractory RCC suggests that some metastatic RCC cells are resistant to mTORC1 inhibition.^21^, ^22^ As shown in Figure 3C, combination of MC‐4 (100 μg/mL) and everolimus (1 μmol/L) dramatically enhanced cytotoxicity in RCC cells. We therefore investigated whether synergistic cytotoxicity by this combination may be caused, at least in part, by the modulation of MC‐4‐induced autophagic cell death by everolimus. Treatment with everolimus affected targets downstream of mTORC2, such as increased phosphorylation of Akt and increased PKM2/GLUT1 expression, in Caki‐1 and 786‐O cells (Figure 3D), indicating that mTORC1 inhibition by everolimus resulted in increased Akt activity through a negative feedback loop. The inhibition of this compensatory feedback by MC‐4 was also supported by synergistic reductions in HIF1α, PKM2, and GLUT1 levels when everolimus and MC‐4 were used in combination (Figure 3D). Although mTOR is hyperactive in VHL‐deficient 786‐O cells compared to Caki‐1 cells with wild‐type VHL expression, our data indicate that MC‐4‐induced cytotoxicity occurs in a VHL‐independent manner via cell cycle arrest, apoptosis, and autophagic cell death. The anti‐proliferative effect of everolimus was due to G1 arrest in Caki‐1 cells and due to G2/M arrest in 786‐O cells (Figure 3E).

Although the combination of MC‐4 with everolimus markedly increased apoptotic cell death in RCC (Figure 3F, left panel), we failed to observe significantly increased cell apoptosis by everolimus and/or MC‐4 in RCC cells, suggesting that apoptosis may not play a significant role in the cytotoxic effects of the combination. These results were confirmed by the absence of a significant change in Bcl‐2/Bax ratio in RCC cells after combination treatment compared with everolimus treatment alone (Figure 3F, right panel). The combination of MC‐4 and everolimus markedly increased autophagosomal formation in human RCCs in vitro, as assessed by MDC staining, and this effect was attenuated by the autophagy inhibitor, 3‐methyladenine (3‐MA),^23^ indicating crosstalk between autophagy and cell metabolism pathways (Figure 3G). Similarly, we observed an increase in cellular lysosomes as well as autophagic vacuoles and empty autophagosomes, presumably with fully digested contents, following exposure to MC‐4+ everolimus in a morphological analysis by TEM (Figure 3H). In addition to its role in anabolic pathways, glutamine metabolism may also promote lactate accumulation and exacerbate glycolysis.^24^, ^25^ Increased anabolic metabolite (lactate) and glutamine utilization via concomitant MC‐4+ everolimus‐induced Akt/PKM2 and mTORC1 pathways could have resulted in marked autophagic cell death (Figure 3I). Therefore, mechanistically, we hypothesize that the modulation of autophagy and cancer cell metabolism by combination treatment with MC‐4 and everolimus, the autophagy inducer, is a powerful approach that could markedly improve therapeutic outcomes in patients with advanced RCC.

### 3.3 MC‐4 combined with everolimus displays potent inhibition of tumor progression in RCC cell xenograft animal models

**Figure 4:**
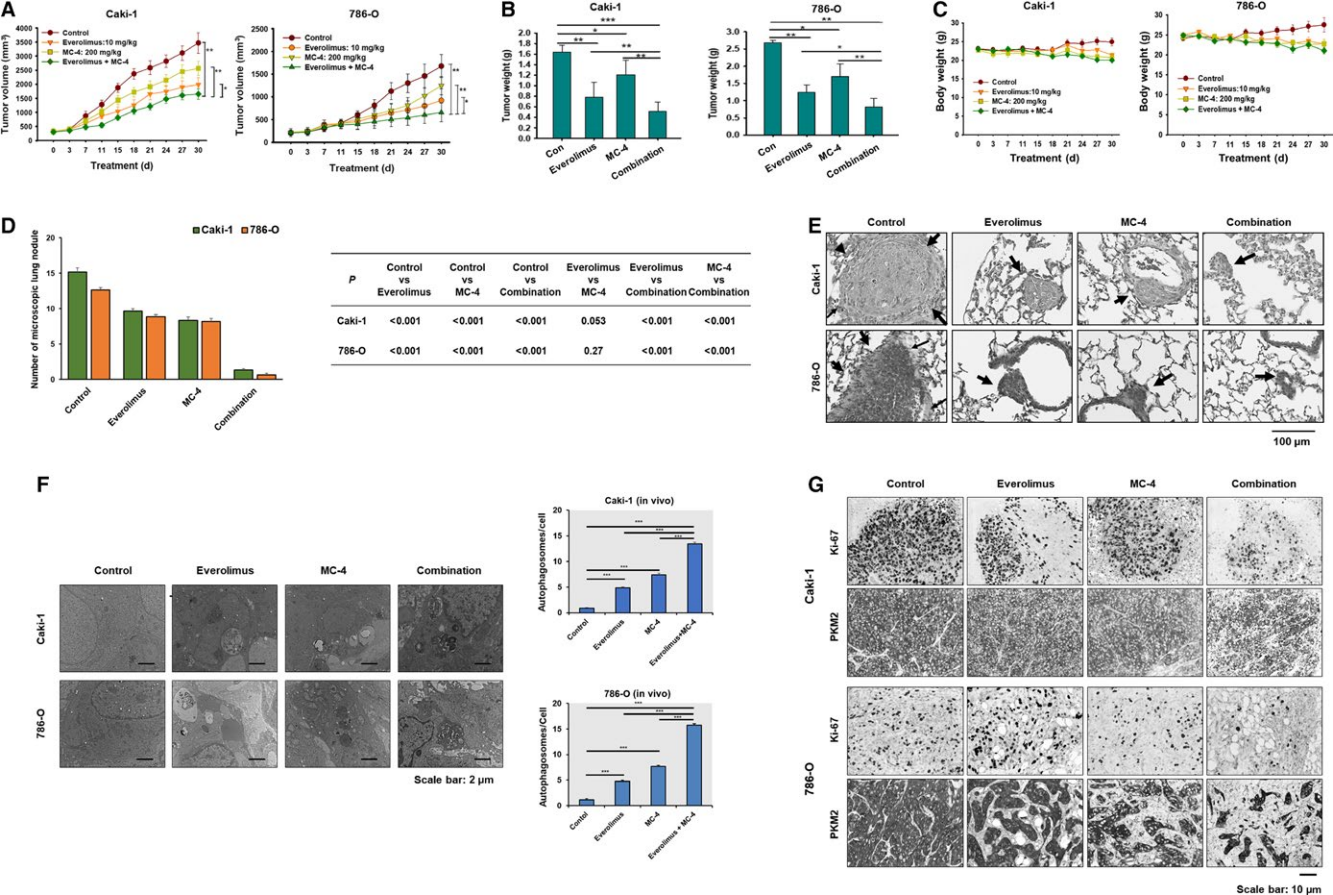
Everolimus in combination with MC‐4 enhances xenograft growth and lung metastasis in human RCC cells. A, MC‐4 and everolimus in combination significantly inhibits RCC tumor growth. Nude mice bearing 100‐mm^3^ tumors were given MC‐4 (200 mg/kg) or everolimus (10 mg/kg) or their combination orally for the days indicated. Tumor size was measured once every 3 d. Each data point represents the mean ± SEM from five mice. *P < 0.05, **P < 0.01. B, The histogram plots show the average tumor weight (mean ± SEM, n = 5). **P <* *0.01 vs vehicle control (Con). C, Effects of MC‐4 and everolimus on the body weight changes in nude mice inoculated with Caki‐1 (left plot) and 786‐O (right plot) cells. There was no statistically significant difference in body weight of the mice among the treatment groups. D, In vivo anti‐metastatic effects of everolimus in combination with MC‐4 (7 mice per each group). All the treatments were performed 5 d a week for 33 d from the day following the injection day. Each data point represents the mean ± SEM. E, Representative illustrations of lung micrometastasis from hematoxylin and eosin stained sections are shown. Arrows indicate lung micrometastasis. Scale bars = 100 μm. F, Representative photomicrographs of immunohistochemical analysis showing that Ki‐67 and PKM2 are reduced in tumors treated with the combination of MC‐4 and everolimus. G, Transmission electron microscopy (TEM) of RCC tumors after treatment with MC‐4, everolimus, or their combination. Histograms are the means ± SEM of two experiments and show the number of autophagosomes/cell in Caki‐1 and 786‐O xenograft tumors. **P <* *0.01, ***P <* *0.001

We evaluated the antitumor activity of the combination of MC‐4 and everolimus in RCC xenograft mouse models to investigate the relevance of coordinate autophagy and mTOR inhibition in RCC tumor growth in vivo. Consistent with the in vitro antitumor effects, treatment with both MC‐4 (200 mg/kg) and everolimus (10 mg/kg) significantly slowed down tumor growth in subcutaneous xenograft mice models of Caki‐1 and 786‐O cells compared with the control (Figure 4A,B). No discernible side effects, such as weight loss (Figure 4C) or change in behavior, were observed. Notably, meaningful reduction in lung metastatic foci was observed after combination treatment with MC‐4 and everolimus in experimental metastasis models of both RCC cells (Figure 4D,E). As shown in Figure 4F, the typical autophagic features of cells were observed after combination treatment with everolimus and MC‐4, whereas untreated cells had normal nuclear and cytoplasmic morphology and significantly lower autophagosomal counts. To investigate whether the suppression of tumor growth by the combination treatment is also associated with decreased proliferation and increased autophagy *in vivo* as observed *in vitro*, we immunostained the tumor sections for Ki‐67 and PKM2 (Figure 4G). Treatment with everolimus and MC‐4, either alone or in combination, led to a reduction in cell proliferation as demonstrated by decreased Ki‐67 staining. Tumors from mice treated with everolimus alone showed a marginal reduction in active PKM2, which was further decreased in tumors from mice treated with the combination (Figure 4G). Furthermore, we confirmed that the synergistic effect of the cotreatment resulted in increased autophagic cell death.

## 4 DISCUSSION

**Figure 5:**
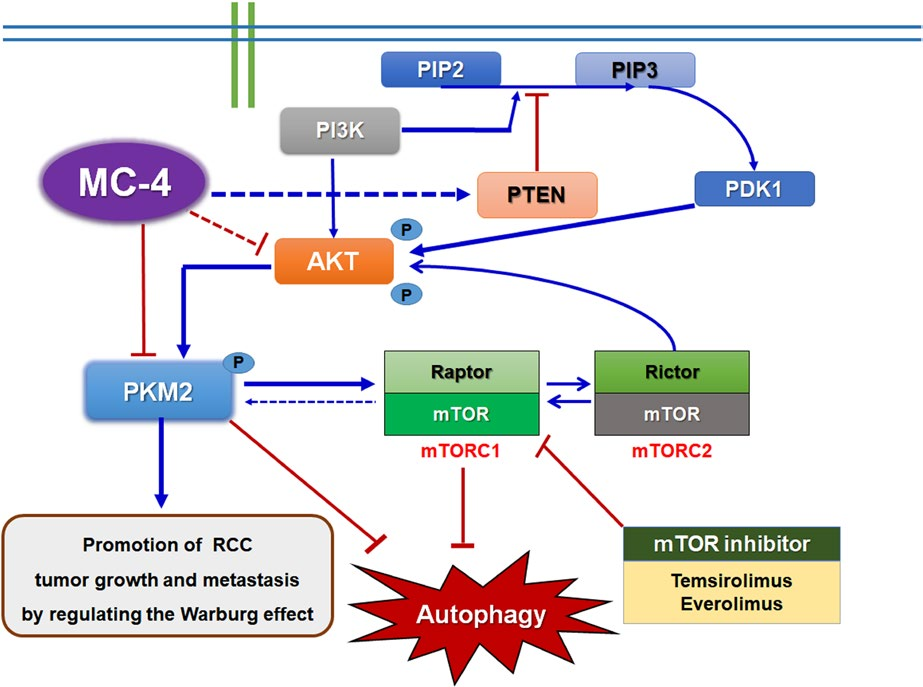
Proposed mechanisms by which PKM2‐mTORC1 regulatory pathways contribute to resistance in RCC cells. Overexpression of PKM2 leads to accumulation of anabolic intermediates and activation of mTOR signaling, which promotes utilization of anabolic intermediates and inhibits autophagy. Meanwhile, mTORC1 activates PKM2 to form a positive loop to enhance the anabolic processes. Signals that activate either PKM2 or mTORC1 can result in anabolic functions. In this pathway, mRCC cells can be sensitized to everolimus by combination treatment with MC‐4 via inhibition of PI3K/PKM2/mTOR signaling pathways

An important and promising target for metastatic RCC to date is mTOR,^4^, ^5^, ^6^, ^7^, ^21^ which is a pivotal regulator of the metabolic pathway of a cell. mTOR receives input from sensors of energy, nutrients, and stress and produces output that regulates protein synthesis and cell growth.^4^, ^5^, ^6^, ^7^ The activation of class I PI3K could lead to activation of Akt‐mTOR signaling pathway, which in turn inhibits autophagy.^26^ However, everolimus monotherapy shows inadequate therapeutic benefits owing to the compensatory activation of mTORC2/Akt signaling, which serves as a key resistance factor.^22^, ^27^, ^28^, ^29^ Therefore, concurrent treatment of metastatic RCC with mTORC1 inhibitors and other targeted agents has been studied to enhance antitumor effects by parallel inhibition of multiple oncogenic signaling pathways. Owing to these concerns, we chose to combine MC‐4 with everolimus to determine whether this combination could potentiate autophagic flux leading to cell death in RCC cells without side effects. The proposed mechanism is summarized in Figure 5. Our results indicate that the simultaneous targeting of tumor cell growth and autophagic cell death by MC‐4 and everolimus results in synergistic antitumor activity mediated by dual inhibition of Akt/PKM2 and mTOR pathways, providing an unexpected opportunity for the development and implementation of drugs targeting cell metabolism and aberrant Akt/PKM2 signaling. Consistent with these results, previous preclinical studies demonstrated that knockdown of PKM2 can inhibit gastric cancer cell proliferation and G1‐S phase transition, attenuate gastric cancer cell migration, and promote autophagy, which may depend on mediating the PI3K/Akt signaling.^30^ These findings suggest that PKM2 might serve as a novel prognostic biomarker and target that would allow for a novel treatment strategy for metastatic RCC in clinical settings. The enhanced antitumor efficacy and autophagic cell death mediated by combination treatment with MC‐4, a novel compound extracted from *Artemisia annua* L., and everolimus via simultaneous inhibition of Akt/PKM2 and mTOR pathways provide a mechanistic rationale to evaluate mTOR inhibitors combined with MC‐4 in clinical studies on patients with metastatic RCC.

**Figure 6:**
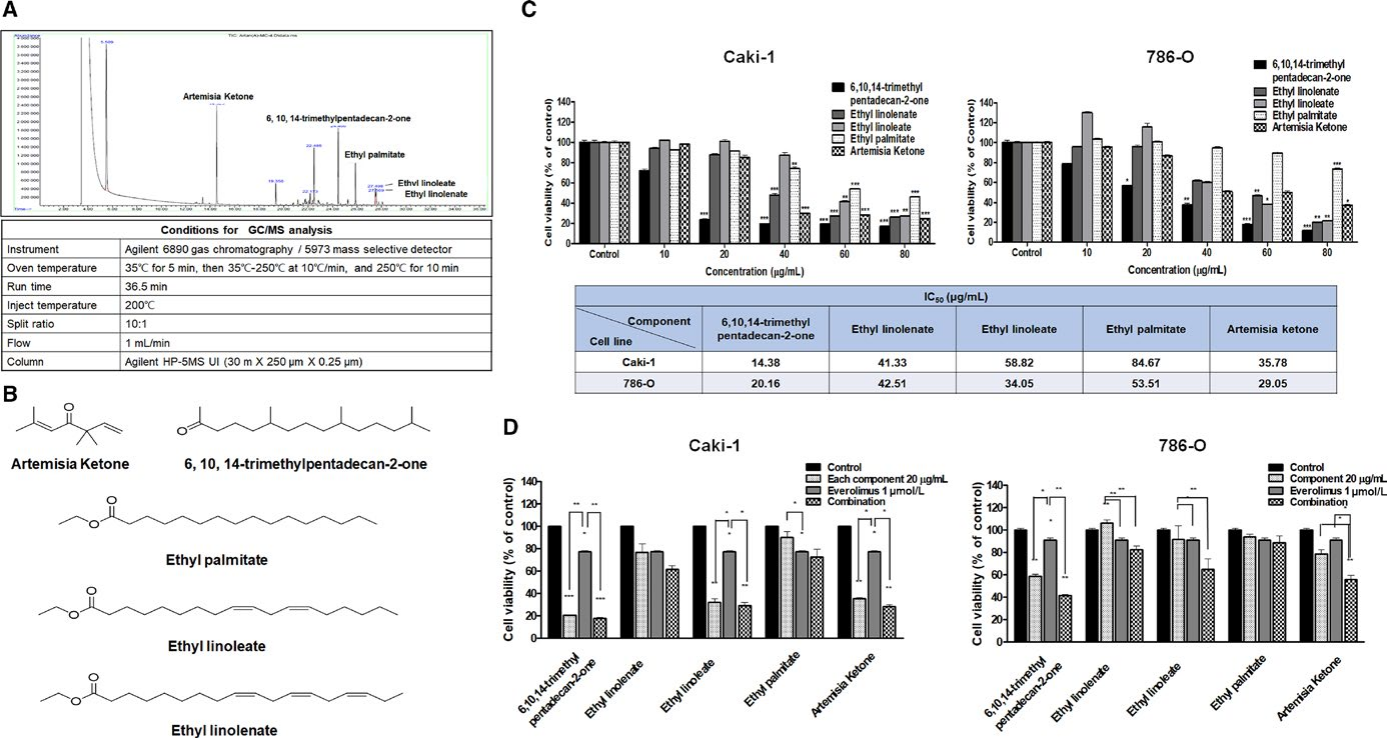
Cytotoxicity of each component isolated from MC‐4 against human RCC cell lines. A, Gas chromatography (GC) chromatogram of MC‐4 and analytical conditions for GC/MS. B, The structures of some of the compounds identified in MC‐4 extract. C, Caki‐1 and 786‐O cells were seeded on 96‐well plates at a final concentration of 2 × 10^3^ cells per 100 μL medium per well at 37°C to allow cell attachment. After 24‐h incubation, the cells were treated with 6,10,14‐trimethyl pentadecan‐2‐one, ethyl linoleate, ethyl linolenate, and ethyl palmitate up to 80 μg/mL for 48 h. Cytotoxicity was determined using MTT assay and the results are expressed as percentages of cytotoxicity relative to the untreated control. Data are expressed as mean ± SEM. *P <* *0.05, **P <* *0.01, ***P <* *0.001. D, Combination effects of each component and everolimus on cell viability. RCC cell lines were treated with MC‐4 in combination with everolimus for 48 h. Cell viability was determined by MTT assay and is expressed relative to the value without treatment. The data are means ± SEM from three independent experiments. **P <* *0.01, ***P <* *0.001

The findings of this study would encourage further clinical studies combining MC‐4 with established chemotherapeutics as a strategy to treat patients with rapidly progressing RCC, regardless of VHL gene mutation status. We focused further on the discovery of novel therapeutics from natural compounds for metastatic RCC and found that MC‐4 consisted of five major components with different cytotoxic effects in RCC cells. The compounds corresponding to five major peaks in GC chromatogram for MC‐4 were identified as artemisia ketone (RT, 14.525 minutes), 6,10,14‐trimethylpentadecan‐2‐one (RT, 24.466 minutes), ethyl palmitate (RT, 25.870), ethyl linoleate (RT, 27.498), and ethyl linolenate (RT, 27.569) from spectroscopic data (Figure 6A,B). As these pure compounds have not been previously reported to have anticancer activity, we compared the potential anticancer activities of the major components in RCC cells. As shown in Figure 6C, 6,10,14‐trimethylpentadecan‐2‐one exhibited the strongest antiproliferative activities on Caki‐1 and 786‐O cells, with an IC_50_ value of 15 μg/mL and 20 μg/mL, artemisia ketone with an IC_50_ value of 36 μg/mL and 20 μg/mL, followed by ethyl linolenate (IC_50_ = 41 μg/mL and 43 μg/mL), ethyl linoleate (IC_50_ = 59 μg/mL), and ethyl palmitate (IC_50_ = 85 μg/mL and 54 μg/mL). Thus, we concluded that the two components, artemisia ketone and 6,10,14‐trimethylpentadecan‐2‐one, exhibiting greater cytotoxicity both in Caki‐1 and 786‐O cells should be evaluated for therapeutic potential against RCC. In addition, the most potent cytotoxicity in Caki‐1 cells and 786‐O cells was induced by combination treatment of artemisia ketone with everolimus (Figure 6D). Thus, the combination of artemisia ketone and everolimus may provide us a new approach to reverse drug resistance in patients with RCC. MC‐4 or each major component (artemisia ketone and 6,10,14‐trimethyl pentadecan‐2‐one) in combination with everolimus showed synergistic cytotoxic effects in RCC cells. We also confirmed the results based on morphological changes and shrinkage of cells leading to cell death induced by each component or MC‐4 in combination with everolimus in RCC cell lines (data not shown). Further investigations into the mechanism of the combination are underway.

Autophagic cell death, an alternative cell death pathway to apoptosis, mediates the antitumor ability of a number of mTOR inhibitors or AMP‐activated protein kinase activators.^31^, ^32^ It mitigates cellular oxidative stress, probably by disposing damaged organelles, especially mitochondria.^33^ Sustained autophagy may eventually cause cell death when protein and organelle turnover exceeds cell capacity.^34^, ^35^, ^36^ For example, dual targeting of autophagic regulation with autophagy enhancers produced therapeutic benefits via cytotoxic autophagic flux in glioma.^34^ As mTORC1 complex is currently considered the autophagy master switch acting like a sensor of nutrient levels to promote proliferation and inhibit autophagy by signaling to the Unc‐51 like autophagy activating kinase 1 (ULK1) complex,^37^ mTORC1 inhibitors have been shown to induce autophagy in tumor cells.^38^, ^39^, ^40^ Pharmacologic blockage of mTORC1 can mimic starvation and promote dissociation of mTORC1 from the complex of ATG13 with ULK1 and ULK2, thereby leaving ULK1‐2 free to initiate autophagy.^38^ Based on these rationales, several early phase clinical trials are ongoing to evaluate the safety and activity of combined autophagy‐mTOR inhibition.^41^, ^42^ In this study, the obvious accumulation of autophagosomes in MC‐4‐treated cells implies that MC‐4‐induced autophagic cell death supported by reduced p62 levels is associated with increased functional autophagic flux in both the human RCC cell lines compared with control cells.^43^ Notably, autophagy was significantly induced by the combination of everolimus and MC‐4, with marked beclin I and LC3‐II activation, indicating that everolimus and MC‐4 synergistically inhibit mTOR activation, which leads to autophagic and not apoptotic cell death.

Increased glutamine metabolism, which is a commonly observed metabolic alteration in cancer cells, promotes lactate accumulation and inhibits glycolysis.^25^, ^44^ However, active mTORC1 and Akt in cancer cells promote the “rewiring” of multiple key nodes of central carbon metabolism to increase glucose flux into glycolysis via the pentose phosphate pathway, thereby ensuring the production of the bioenergetic (ATP) and anabolic requirements of cellular growth and proliferation.^45^, ^46^ In particular, Akt/mTORC1 activation upregulated PKM2, which catalyzes one of the final steps of glycolysis by dephosphorylating phosphoenolpyruvate to pyruvate.^47^ Recently, PKM2 has been shown to play an important role in the reprogramming and maintenance of altered metabolism in cancer cells and has also been demonstrated to directly regulate gene expression and subsequent cell cycle progression.^48^, ^49^ Furthermore, inhibition of mTOR pathway reduces glucose uptake by downregulating GLUT1 activity.^46^ Therefore, co‐targeting mTORC1/Akt and glycolysis via GLUT1/PKM2 prevents metabolic rewiring and may lead to better antitumor activity, and GLUT1 and PKM2 dependency might explain the lack of response to agents targeting these processes in some population of cancer patients.^50^ We demonstrated that MC‐4 induced autophagic cell death rather than apoptosis in RCC cells possibly by inhibiting glucose metabolism via a marked decrease in PKM2 and GLUT1 expression modulated by the Akt/PKM2 pathway. As mTORC1 inhibitors such as everolimus activate Akt, the co‐administration of a PKM2 inhibitor, such as MC‐4, might help overcome this effect and lead to greater cell killing than that observed with mTORC1 inhibition alone. Overall, the present study shows for the first time that PKM2 inhibitors can potentiate the antitumor effects of mTOR1 inhibitors in RCC. Notably, although mTOR is hyperactive in VHL‐deficient 786‐O cells compared with that in Caki‐1 cells with wild‐type VHL expression, both RCC cell types showed similar sensitization to everolimus in the presence of MC‐4, indicating VHL‐independent phosphoinositide 3‐kinase /PKM2/mTOR signaling inhibition. These findings reveal the potential therapeutic benefits of clinical application of MC‐4 with a lower dose of mTOR inhibitors for metastatic RCC patients by modulating cancer cell metabolism and autophagy.

## CONFLICT OF INTEREST

No conflicts of interest, including specific financial interests, relationships, and affiliations relevant to the subject matter or materials discussed in the manuscript were disclosed.

## AUTHOR CONTRIBUTION

J. Y. Son and S. Yoon are co‐first authors. J. Y. Son, S. Yoon, I. H. Tae, Y. J. Park, U. De, Y. Jeon, Y. J. Park, I. J. Rhyu, B. M. Lee, K. ‐H. Chung, and S. J. Lee performed most of the experiments and analyses. J. H. Kwak, H. W. Lee, H. S. Kim, and H. Y. Choi wrote the manuscript and organized the figures. H. W. Lee, H. S. Kim, and H. Y. Choi designed and supervised the entire project.
